# Acute HIV infection presenting as hemophagocytic lymphohistiocytosis: case report and review of the literature

**DOI:** 10.1186/s12879-017-2732-y

**Published:** 2017-09-20

**Authors:** Farheen Manji, Evan Wilson, Etienne Mahe, John Gill, John Conly

**Affiliations:** 1 University of Calgary and Alberta Health Services, 1403-29th Street NW, Calgary, AB T2N 2T9 Canada; 2 Foothills Medical Centre, Alberta Health Services-Calgary and Area, Room AGW5, Foothills Medical Centre, 1403-29th Street NW, Calgary, AB T2N 2T9 Canada

**Keywords:** Hemophagocytic syndrome, HIV, Human immunodeficiency virus, Acute retroviral syndrome, Hemophagocytic lymphohistiocytosis

## Abstract

**Background:**

Hemophagocytic lymphohistiocytosis (HLH) is an uncommon systemic inflammatory condition that can result from infections, autoimmune diseases and malignancies. It is a rarely reported life threatening complication of an acute HIV infection, with only ten documented case reports per our literature search. We present a case of HLH secondary to acute HIV infection with a negative HIV antibody-based assay and high plasma viral load.

**Case presentation:**

A 45 year old male with a past medical history of well controlled hypertension presented with fever, dizziness and non-bloody diarrhea. Initial lab work revealed a new thrombocytopenia, marked renal failure and an elevated creatine kinase, ferritin, lactate dehydrogenase and D-dimer. A bone marrow biopsy revealed HLH. As part of the work up for thrombocytopenia, a rapid HIV antibody based assay was done and was negative. The sample was later routinely tested with a fourth generation antigen/antibody assay as per local protocol and was strongly positive. The plasma RNA viral load was >10,000,000 copies /mL confirming the diagnosis of an acute HIV infection. The patient was urgently started on antiretroviral therapy and recovered.

**Conclusion:**

This case illustrates a diagnostic approach to HLH which is an uncommon but life threatening multisystem disease, requiring the involvement of a multidisciplinary team of experts. Following any diagnosis of HLH, rapid identification and treatment of the underlying condition is critical. A negative rapid HIV antibody test can be misleading in the context of early HIV infection and the additional use of fourth generation antigen/antibody test or plasma RNA viral load may be required within the right clinical context for diagnosis.

## Background

Hemophagocytic lymphohistiocytosis (HLH) is rare and life-threatening immune activation syndrome that can be idiopathic or secondary to various infectious, inflammatory or neoplastic conditions (Table 1) [1–3]. HLH associated with HIV is rare, most often being described in those with chronic HIV infection or with the presence of concomitant opportunistic infections [4]. HLH associated with an acute HIV infection is even rarer, having been described in only a few case reports [5–12]. We report the case of a 45 year old male with life-threatening HLH secondary to an acute HIV infection and a negative rapid antibody-based HIV test. HIV was diagnosed based on a fourth generation antigen/antibody based Enzyme Linked Immunoassay and plasma RNA viral load. The patient recovered with antiretroviral therapy (ART). In addition, we review the available literature on this topic.

**Table 1:** Differential diagnosis of secondary hemophagocytic lymphohistiocytosis [1]

Infectious	
Viral	Epstein-Barr Virus, Cytomegalovirus, Parvovirus, Herpes Simplex Virus, Varicella-Zoster Virus, Measles, Human Herpes Virus-8, H1N1 Influenza Virus, Parechovirus, HIV
Bacterial	*Brucella spp.,* Gram negative bacteria, *Mycobacterium tuberculosis*, *Staphylococcus aureus*,
Fungal	*Candida, Cryptococcus, Pneumocystis, Histoplasma, Aspergillus*
Parasitic	*Plasmodium falciparum*, *Plasmodium vivax*, *Toxoplasma*, *Leishmania*, *Strongyloides*
Inflammatory	Systemic juvenile idiopathic arthritis, Kawasaki’s disease systemic lupus erythematosus and other rheumatologic diseases
Neoplastic	T-cell/NK-cell lymphomas, anaplastic large cell lymphomas, acute lymphoblastic leukemia, Hodgkin’s lymphoma, various solid tumours (prostate, lung, hepatocellular carcinoma)
Immunodeficiencies	Inherited or acquired immunodeficiency

## Case presentation

A middle aged man with a history of controlled hypertension presented with a one week history of non-bloody diarrhea with fever and dizziness. He reported four to six non-bloody bowel movements a day for the one week prior to admission with subjective fevers and a sore throat. He reported being in a monogamous relationship with his wife and denied use of any illicit intravenous or oral drugs, alcohol or recent travel outside Canada.

Examination revealed a fever of 38.0 °C, heart rate of 76, blood pressure of 163/100 and a respiratory rate of 16 with saturations of 93% on room air. He had erythematous tonsils with no exudate and a clinical diagnosis of oral candidiasis. There was no rash, palpable lymphadenopathy or oral ulcers but he had mild bleeding of his gums. He had a fluctuant mass on his left upper thigh with purulent drainage. The rest of his exam was unremarkable.

The laboratory results are shown in Table 2. He was found to have a marked thrombocytopenia and lymphopenia and an elevated creatinine with granular casts in his urine and a markedly elevated creatine kinase (CK). There was no evidence of hemolysis but he did have a high lactate dehydrogenase, ferritin and D-Dimer. The peripheral blood smear showed thrombocytopenia with no evidence of schistocytes. He had mild splenomegaly (13.3 cm, normal <12 cm) on ultrasound. On his third day of admission, one out of two sets of blood cultures was positive for methicillin-resistant *Staphylococcus aureus* (MRSA) at 22 h and two out of two blood cultures were positive for *Streptococcus mitis* (*S.mitis*) at 14 h. The MRSA strain had a susceptibility profile typical for a community associated MRSA. Syphilis EIA testing and urine gonorrhea and chlamydia assays were negative. Anti-nuclear antibodies and C3 and C4 were negative. Hepatitis A IgG, was reactive, hepatitis B core antibody/antigen, and hepatitis B surface antigen were negative while the surface antibody was positive at 1.2 IU/L. Hepatitis C core antibody was non-reactive. Epstein-Barr Virus (EBV) IgM antibodies were negative and IgG antibodies were positive, consistent with a remote infection. Parvovirus B19 IgM and CMV IgM were negative. A rapid HIV antibody based assay (INSTITM HIV-1/HIV-2 Antibody Test Kit, bioLytical Laboratories Inc.) was negative.

**Table 2:** Laboratory values at presentation and after treatment

	Patient lab values		Normal range
	At admission	After 5 weeks of antiretroviral therapy	
Hemoglobin, g/L	159	117	137–180
White blood cell, ×10^9^/L	3.0	3.2	4.0–11.0
Platelets, ×10^9^/L	50	207	150–400
Sodium, mmol/L	139	139	133–145
Potassium, mmol/L	3.4	4.0	3.3–5.1
Chloride, mmol/L	107	108	98–111
Bicarbonate, mmol/L	16	24	21–31
Creatinine, μmol/L	160	97	50–120
Total bilirubin, μmol/L	20	11	0–24
Haptoglobin, g/L	1.24		0.30–2.00
Lactate Dehydrogenase (LDH), U/L	1739	242	100–235
Ferritin, μg/L	69,717		30–400
D-dimer, mg/L	>10		<0.46
Fibrinogen, g/L	2.0		1.6–4.1
Creatine kinase, U/L	25,000	232	0–195
Triglycerides, mmol/L	4.36		0.60–2.30
CD4 Count, ×10^9^/L	0.137	0.275	0.499–1.651
Viral load, copies/mL	>7.24 log base 10	1.96 log base 10	
HIV Architect 4th generation assay	615.18	27.3	<1
Western blot	Negative		Negative

**Fig. 1:**
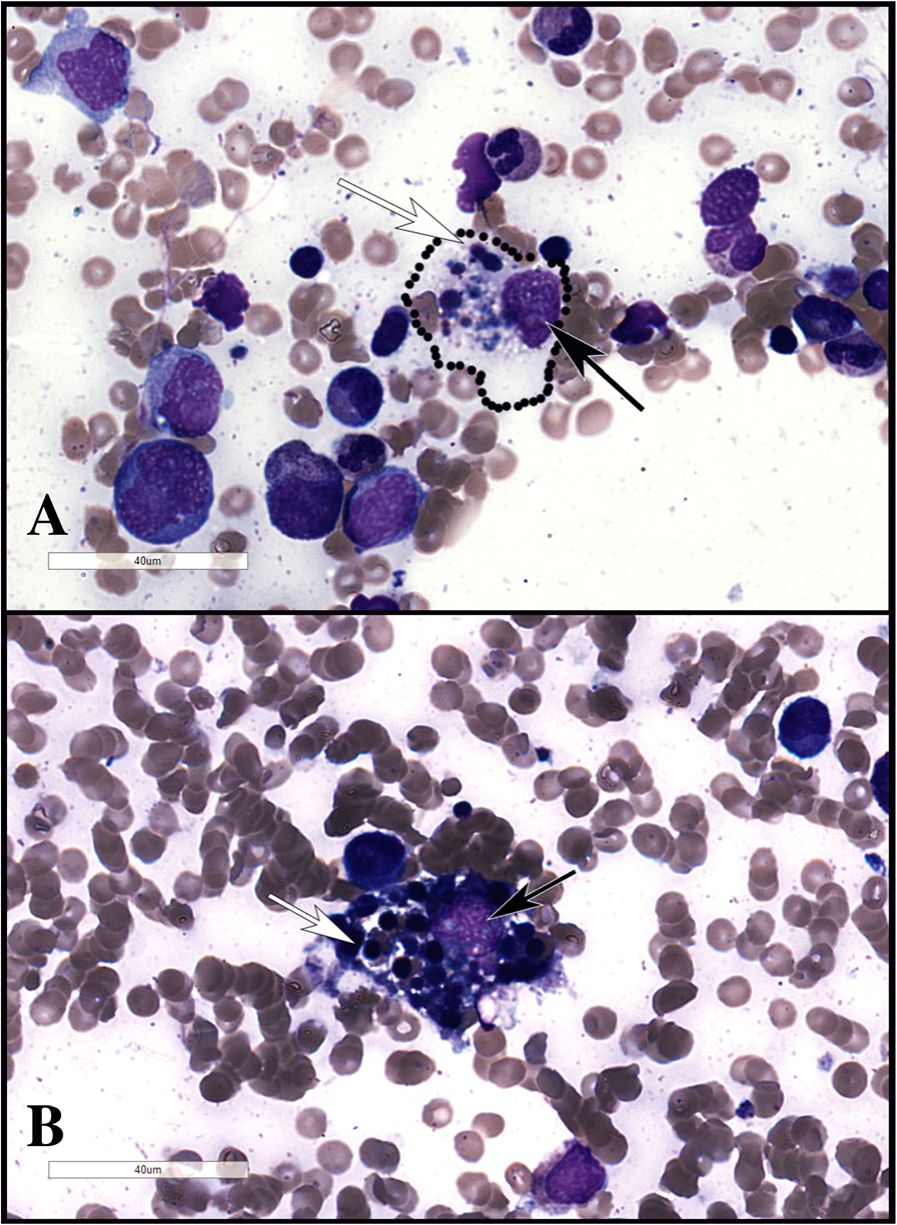
A compilation of two photomicrographs from the bone marrow aspirate with a Giemsa stain. In both images **a** and **b**, high-power views of hemophagocytic histiocytes are shown, with the histiocyte nucleus highlighted by the black-colored arrow and the partially-digested nuclei of phagocytosed cells highlighted by the white-colored arrow. The cytoplasmic border of the histiocyte of interest in image A is highlighted by the dotted line. Giemsa staining is performed using air dried aspirate smears

**Fig. 2:**
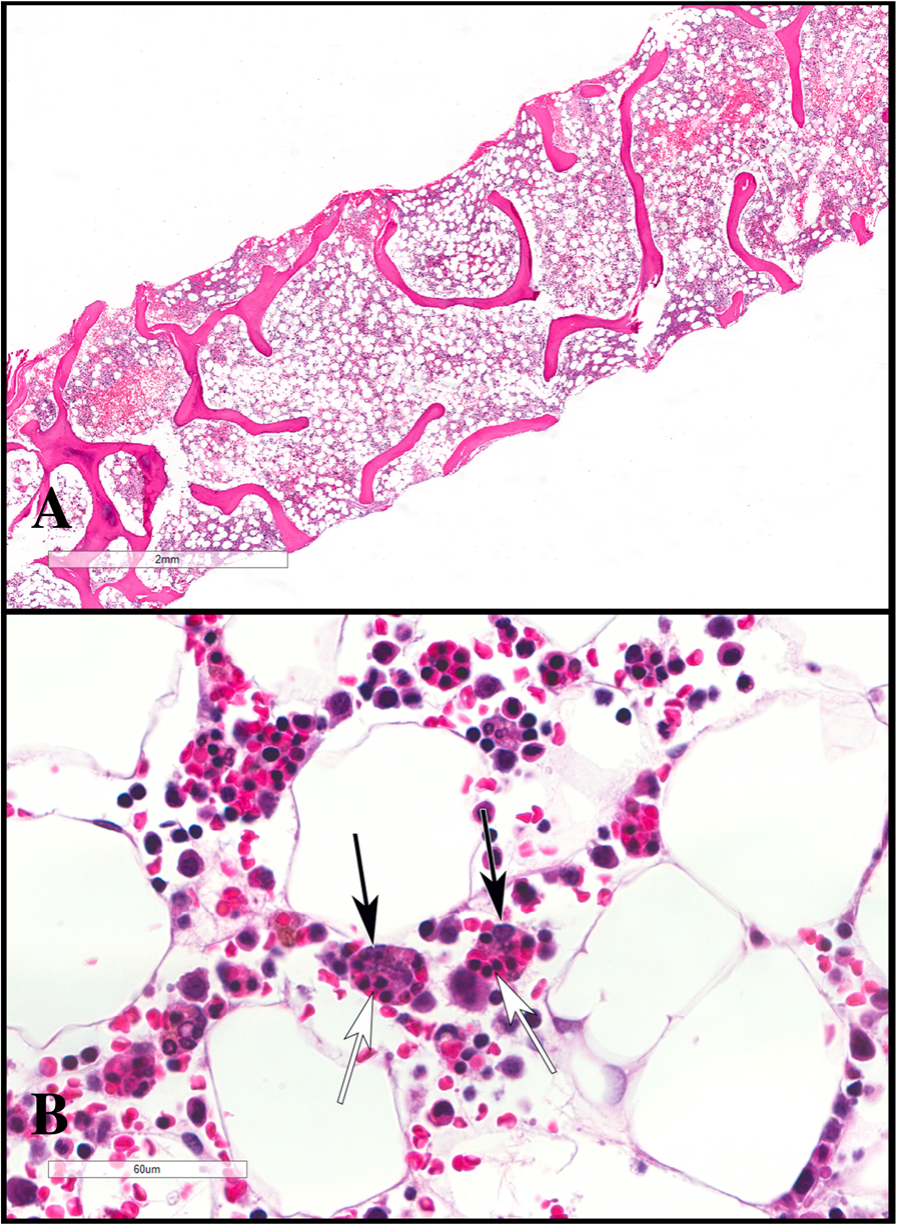
A compilation of two photomicrographs from the bone marrow biopsy with a Hematoxylin/Eosin (H&E) stain. In image **a**, a low-power image, the hypocellularity of the marrow (considering the patient’s young age) is highlighted. In image **b**, a high-power image, several hemophagocytic histiocytes can be seen. Both have a saccular appearance (their nuclei are highlighted by the black-colored arrow), dilated by phagocytosed marrow elements (whose partially-digested nuclei are highlighted with the white-colored arrow). H&E biopsy stains are prepared on formalin-fixed paraffin embedded bone marrow biopsy materials that are briefly decalcified in formic acid

Bone marrow biopsy revealed a hypocellular marrow with multifocal hemophagocytosis with no evidence of a neoplastic, parasitic or fungal process (Figs. 1 and 2).

The sample submitted for rapid HIV testing was later routinely tested with a fourth generation antigen/antibody assay (Abbott Architect, Abbott Illinois USA) as per local protocol. The test was strongly positive with a signal to cut off ratio of 615.18 (Normal <1) and the Western blot showed no antibody bands. The plasma RNA viral load was >10,000,000 copies/mL (Abbott Realtime Illinois USA), confirming the diagnosis of acute HIV infection. His CD4 count was low at 0.137 × 10^9^/L (normal 0.499–1.651 × 10^9^/L).

A diagnosis of acute HIV infection with secondary HLH was made based on criteria from the HLH-2004 trial [1]. A secondary diagnosis of acute kidney injury secondary to rhabdomyolysis occurring in association with the acute HIV seroconversion illness was also made. The MRSA bacteremia was secondary to a subcutaneous abscess in his upper left thigh and the *S. mitis* bacteremia was thought to be secondary to the bleeding gums as a portal of entry. He was immediately started on an anti-retroviral therapy (ART) regimen consisting of lamivudine 300 mg once daily, dolutegravir 50 mg once daily and rilpivirine 25 mg once daily,based on his overall findings, local resistance patterns to first generation non-nucleoside reverse transcriptase inhibitors and the desire for rapid drop in viral load given the underlying manifestations of his HIV infection.

After five weeks of ART, the plasma RNA viral load had decreased to less than 100 copies/mL and his CD4+ count had increased to 0.275 × 10^9^/L. His platelets, CK, ferritin and creatinine normalized. He responded well to two weeks of intravenous vancomycin and remained adherent to ART. The risk factor for HIV infection remains unclear. By three months follow up, he had developed a full band profile of antibodies to HIV on Western blotting, the viral load was undetectable and all other results had normalized.

## Discussion

Acute HIV infection may be asymptomatic in about half of patients [13]. Symptomatic acute HIV infection presents with nonspecific features such as fever, pharyngitis, lymphadenopathy, a diffuse maculopapular rash, myalgia and malaise although other atypical presentations have been described in the literature [14].

The diagnosis of HLH can be made based on criteria from the HLH-2004 trial but is difficult to diagnose clinically with a broad differential diagnosis including infections, neoplasms, inflammatory disorders and immunodeficiencies (Table 1) [1].

HLH is a rare presentation of an acute HIV infection; based on a literature search of articles in English, we were able to find 10 documented cases of adults who presented with HLH secondary to an acute HIV infection [5–12]. Due to its rarity, diagnosis is often difficult, delaying treatment. In this case, a markedly elevated ferritin increased suspicion for HLH which, along with the thrombocytopenia, prompted a bone marrow biopsy. As part of the work up for an underlying cause of HLH, a rapid HIV assay was ordered which was negative. All HIV antibody tests are known to miss the “window period” of acute HIV infection between infection and antibody production [15]. As such, fourth generation antigen/antibody assays along with plasma viral load must be used for diagnosis as per Centers for Disease Control and Prevention guidelines [16].

In cases of secondary HLH, no immunomodulators are used but rather treatment of the primary condition is given to control the acute inflammatory cascade that results in HLH. We used no immunomodulatory therapy and chose, in the absence of resistance testing, an empiric regimen to suppress viremia rapidly in an effort to reduce the active HLH related co-morbidities.

This case report suggests that acute HIV infection needs to be considered when the cause of HLH is not apparent. While rapid HIV antibody testing is fast and easily accessible, its limitations in diagnosis of early HIV need to be acknowledged as it cannot be used to exclude acute infection prior to the antibody response. The value of solid testing algorithms and use of fourth generation antigen-antibody assays with molecular testing for a definitive diagnosis are affirmed by this case. With HLH, consideration of all its etiologies with appropriate diagnostic testing is required. While the differential for secondary HLH is broad, consideration of the clinical context can help dictate what kind of testing needs to be done.

## Conclusion

HLH as a presentation of acute HIV infection is rare and potentially life threatening. HLH is a complex multisystem disease and requires the involvement of different disciplines including experts in the fields of infectious diseases, hematology, immunology and pathology for diagnosis and management. Following any diagnosis of HLH, rapid identification and treatment of the underlying condition is critical. Our case suggests that a negative rapid HIV antibody test can be misleading in the context of early HIV infection and the additional use of fourth generation antigen/antibody test or plasma RNA viral load may be required within the right clinical context for diagnosis.

## Abbreviations

ART: Antiretroviral therapy
CK: Creatine kinase
HIV: Human immunodeficiency virus
HLH: Hemophagocytic lymphohistiocytosis
